# The Influence of Communication Modality on the “Saying-Is-Believing” Effect

**DOI:** 10.3390/bs15050639

**Published:** 2025-05-08

**Authors:** Rui Yin, Xianyun Liu

**Affiliations:** 1 Faculty of Psychology, Tianjin Normal University, Tianjin 300387, China; 2230340024@stu.tjnu.edu.cn; 2 Key Research Base of Humanities and Social Sciences of the Ministry of Education, Academy of Psychology and Behavior, Tianjin Normal University, Tianjin 300387, China; 3 Tianjin Key Laboratory of Student Mental Health and Intelligence Assessment, Tianjin 300387, China

**Keywords:** internet, communication modality, “saying-is-believing” effect

## Abstract

In communication, people adjust their information expression based on the audience’s attitude toward a topic, which is known as the audience-tuning effect. This effect also leads individuals to develop memory biases favoring the audience’s attitude, a process termed the “saying-is-believing” (SIB) effect. This study validates the SIB effect using a classical paradigm based on shared reality theory. Additionally, it explores the impact of different communication modalities on the SIB effect, considering the information dissemination context in the internet era and the unique characteristic of “visual anonymity” in online communication compared to offline communication. A two-factor mixed experimental design with 2 (audience’s attitude: positive, negative) × 2 (communication modality: online, offline) was employed. The following results were found: (1) The SIB effect exists, meaning that people adjust their descriptions and recalls based on the audience’s attitude. (2) Communication modality and the audience’s attitude interactively influence the SIB effect, with a greater deviation in description and recall valence when the audience’s attitude is negative (positive) in online (offline) compared to offline (online) communication. In summary, online communication is more likely to generate negative information than offline communication. This study enriches and expands the research field of the SIB effect, filling the gap in cross-media comparisons within this field. Moreover, it further enhances individuals’ understanding of online and offline communication modalities, which has certain guiding significance for enhancing work and learning effectiveness, improving the internet environment, and supporting enterprise management. Future research can further subdivide communication modalities, improve the classical paradigm to make it more practical, and incorporate neural technologies to delve deeper into the influencing factors and underlying mechanisms of the SIB effect.

## 1. Introduction

Communication, as a fundamental human behavior, constitutes the cornerstone of social interaction and interpersonal relationship building. With the rapid development of information technology, profound changes have occurred in communication modes, with online and offline communication coexisting and jointly shaping the complex landscape of information dissemination. According to a report jointly released by the China Internet Network Information Center (CNNIC) in Beijing on 23 December 2023, the internet penetration rate among minors in China reached 97.2% in 2022. An overseas survey study also found that 90% of European adolescents use the internet at least once a day (Eurostat, 2016). Compared to offline communication, online communication offers numerous convenience features, such as higher anonymity, more opportunities to form new social relationships across geographical distances, and faster information dissemination speed (i.e., social media posts can reach thousands of people in a short period of time, which is difficult in offline communication) (Lieberman & Schroeder, 2020).

In addition to the aforementioned characteristics of online communication, studies have also found that individuals may exhibit different behavioral patterns in offline and online environments, particularly as people are more likely to engage in behaviors online that they would not in real life (S. Zhou et al., 2023). Research by Gross (2004) and Valkenburg et al. (2005) indicates that approximately 50% of internet users disguise their identities when communicating online. Caspi and Etgar’s (2023) discussion on emotional issues in online communication revealed that due to the filtering of communication cues by online media, people are more prone to amplifying their emotions, leading to common examples such as blindly following trends in social media comments and unconsciously internalizing others’ attitudes toward focal figures or objects into one’s own memory. Given the complex and diverse nature of today’s online environment, important questions arise: Are people more susceptible to the influence of online attitudes during online interactions, leading to deviations in information output and even affecting their own memory? How should one weigh the pros and cons of online versus offline communication? These are significant topics worthy of in-depth exploration in recent years. The “saying-is-believing” (SIB) effect (Ding & Zheng, 2011; Higgins & Rholes, 1978) and its paradigm provide a powerful perspective for studying these phenomena. Through this perspective, one can delve into how audience’s attitudes influence the information transmission and cognitive processing of speakers across different communication modalities, thereby revealing the psychological mechanisms behind interpersonal communication in various communication modalities and providing a theoretical basis and practical guidance for solving the aforementioned practical problems.

This research has two purposes. First, this study seeks to validate the “saying-is-believing” (SIB) effect in the context of Chinese culture based on the shared reality theory in conjunction with the classic experimental paradigm of the SIB effect. In addition, we explored the influence of different communication modalities on the SIB effect in the context of information dissemination in the internet era and the unique feature of “visual anonymity” of online communication compared to offline communication. Therefore, a two-factor mixed experimental design of 2 (audience’s attitude: positive, negative) × 2 (communication modalities: online, offline) was employed, focusing on the interaction between audience’s attitude and communication modalities.

Our research makes several contributions to the literature. First, this research enriches and expands the research field of the “saying-is-believing” (SIB) effect, and it is the first cross-media comparative study of the “saying-is-believing” (SIB) effect. Second, this study extends the classic SIB paradigm to real-life interaction contexts, examining related issues of the SIB effect within actual temporal and spatial dimensions. Third, this study conducts a deeper analysis of online communication, combining previous research to propose that visual anonymity in online communication exacerbates the amplification effect of audience’s attitude differences on description bias and memory bias through “deindividuation”. It was found that the SIB effect was more likely to occur online compared to offline in the negative condition and offline compared to online in the positive condition. This result has some guiding significance for improving work-study effectiveness, rectifying the internet environment, enhancing and enterprise management. In conclusion, this study introduces innovative variables and paradigms on the basis of previous theories and offers some practical significance in the field of internet and enterprise management.

## 2. Literature Review

### 2.1. The SIB Effect and the Shared Reality Theory

The “Saying-is-Believing” Effect (SIB Effect) originates from individuals’ dynamic adjustments in interpersonal communication based on the audience’s attitude (Ding & Zheng, 2011). These adjustments manifest primarily in two aspects: individual behavior (descriptions) and individual judgments (recall). Higgins and Rholes (1978) first confirmed using a classical experimental paradigm that when a speaker speaks within a social group, they adjust their information presentation on a given topic according to the characteristics of their audience, including their knowledge level, intentions, and attitudes. This phenomenon is known as the audience-tuning effect (Higgins, 2013). Furthermore, the audience-tuning effect can even influence the speaker’s subsequent memory, resulting in a memory bias that favors the audience’s attitude, termed the audience-tuning memory bias (Echterhoff et al., 2005). For instance, when the audience holds a negative attitude toward a target individual, the speaker will use more negative vocabulary in their description and reinforce this negative impression when recalling the event.

This classic experimental paradigm typically uses ambiguous information about a target individual as the communication theme, and its core procedure consists of the following steps. Firstly, participants are required to read a profile of the target individual that includes neutral descriptions. Subsequently, researchers “casually” inform the participants (speakers) about the attitude (positive or negative) of a non-present audience member toward the target individual. Todorov (2002) pointed out that participants’ evaluative judgments are only influenced by the audience’s attitude when the attitude is presented indirectly; if the presentation is too direct, participants may regard it as an external situational cue and actively revise their judgments. Next, the participants, as speakers, need to summarize the characteristics of the target individual to help the audience accurately identify the target from a pool of candidates. Finally, after engaging in an unrelated interference task, participants are asked to recall the information about the target individual from the original learning material as accurately as possible. Numerous previous studies have employed the classic paradigm and its modifications to confirm the ubiquitous presence of the SIB effect (Echterhoff et al., 2017; Knausenberger et al., 2019; Liang et al., 2021; Mata & Semin, 2020; Rossignac-Milon & Higgins, 2018; Ye et al., 2021).

The research scope of the SIB effect has continually expanded with interdisciplinary development, and its universality has been widely verified across cultural, group, and multi-domain contexts. At the level of cognition and memory research, existing studies have revealed the consistency of the SIB effect between Eastern and Western cultures (Liang et al., 2021; Ye et al., 2021). Recent findings have further deepened the exploration of its mechanisms. Wagner et al. (2024) confirmed the critical role of cognitive accessibility in the SIB effect through an analysis of a large age-span sample of individuals aged 18–60 in Germany; Zang et al. (2023), with Chinese college students and depressive populations as subjects, not only verified the universality of the effect but also extended its applicability to the field of mental health. Diversity in research themes has also emerged as a new trend. Traditional paradigms focused on the evaluation of “target individuals”, while current research has gradually extended to electronic word of mouth (eWOM) in consumer behavior and economic decision-making (Anufriev et al., 2023). For instance, the phenomenon of “brand love” built by consumers through shared brand knowledge (Yang et al., 2014) and the verification of the SIB effect in commodity information and scientific communication (Knausenberger et al., 2019) both demonstrate the high contextual flexibility of the effect. However, significant limitations still exist in current research. At the methodological level, the vast majority of experiments follow a single paradigm—that is, speakers conveying written information to fictional audiences (Conley et al., 2016; Echterhoff et al., 2017; Knausenberger et al., 2019; Liang et al., 2021; Pierucci et al., 2014). Although this design can effectively control irrelevant variables, it also leads to systematic neglect of interactive contextual elements (such as non-verbal cues, real-time feedback) and individual trait factors (such as personality tendencies, social motives). Furthermore, in the context of the digital age, the differentiated impacts of online anonymity (Caspi & Etgar, 2023) and offline social cues (Schwartz et al., 2024) on the SIB effect have not been fully explored, and cross-media comparative research remains a gap.

The theory of shared reality effectively explains the audience-tuning effect and its mechanism of influence on memory (Shared Reality, Conley et al., 2016; Echterhoff et al., 2017; Knausenberger et al., 2019; Mata & Semin, 2020; Semin, 2018). Shared reality refers to the process through which individuals experience commonalities in their internal states with others (Echterhoff et al., 2009). According to the shared reality theory, the construction of shared reality is driven by individual intrinsic motivations. On the one hand, individuals aim to enhance their connections with others by sharing and perceiving the world together (relational motivation) (Echterhoff & Higgins, 2019); on the other hand, individuals seek to validate their attitudes, judgments, or feelings about the world through shared experiences with others, thereby transforming their subjective experiences of perceiving the world into objective truths (epistemic motivation) (Wagner et al., 2024). With the development of the shared reality theory, research has further expanded its application scope, extending the audience from a single individual to multiple individuals and broadening shared reality to areas such as racial prejudice (Yantis et al., 2025), socialization in higher education (Pinelli & Higgins, 2024), prosocial behavior (F. Zhou et al., 2024), and disaster trauma (Leshem et al., 2025), providing a theoretical basis for understanding individuals’ attitudes, beliefs, memories, and decisions in information dissemination. Additionally, research has expanded the communication themes of the SIB effect from a single theme to multiple themes, proposing the concept of generalized shared reality (Rossignac-Milon et al., 2021). In 2024, Rossignac-Milon et al. also provided new quantitative assessment standards for shared reality research through standardized measurement methods. In addition to the Generalized Shared Reality (SR-G, Rossignac-Milon et al., 2021) questionnaire, the Self-Report Questionnaire of Shared Reality (SR-T, Rossignac-Milon et al., 2024) was also included in the measurement methods, confirming a significant correlation between the shared experiences of speakers and audiences and memory biases.

### 2.2. Communication Modality and Visual Anonymity

With the rapid evolution of internet technology, online communication has permeated all aspects of our lives. Numerous studies have explored the characteristics of online communication, interpersonal relationships, and self-expression. In 1999, Garrison introduced the concept of social presence into research on computer-mediated interpersonal communication, explaining how people perceive and evaluate the extent and quality of their interactions with others through computer media and communication technologies. (Garrison et al., 1999) Online environments, lacking visual cues (such as facial expressions and body language) and physical presence found in face-to-face interactions, often reduce social constraints through anonymity, enhance social presence, and prompt individuals to engage in deeper self-disclosure or negative expression (Pouwels et al., 2021). Visual anonymity is considered a key influencing factor. Research by Baccon et al. (2019) found that visual anonymity serves as a protective factor in online communication, with speakers disclosing more frequently when visually anonymous. In other words, online social interactions may overcome the limitations of offline social interactions due to the concealment of facial cues, creating opportunities for intimate disclosures and emotional expression (Yau & Reich, 2018). This contributes to the quality of friendships between both parties, primarily by enhancing intimacy (Pouwels et al., 2021; Uhls et al., 2017). The latest research by Hsieh et al. (2024) found that individuals with high (or low) levels of social anxiety tend to choose visually anonymous (or non-anonymous) media for initial interactions. Interestingly, once engaged in visually anonymous media, individuals with high social anxiety can improve their social anxiety by trusting the other party and gradually adopting richer media. This further confirms that visual anonymity is a protective factor, allowing people to relax and gradually unleash their instinctive selves. Mýlek et al. (2024) found that although social anxiety in adolescents directly inhibits offline communication, it increases the frequency of self-disclosure with online strangers through the indirect effect of “Preference for Online Social Interaction” (POSI). Additionally, studies have shown that online communication can also regulate emotions compared to offline communication (Syrjämäki et al., 2024). This contradictory effect stems from the controllability and anonymity of online environments, making it easier for anxious individuals to overcome psychological barriers in real-life social interactions, supporting the “social compensation hypothesis” (Mýlek et al., 2024). Thus, it can be summarized that due to the protection provided by visual anonymity, individuals are more willing to engage in intimate disclosures in online communication.

Based on recent advancements in online communication research, this study integrates multi-dimensional findings and proposes an innovative theoretical model. Previous studies have shown that the strong anonymity of the internet makes it easier for people to engage in undesirable behaviors compared to offline settings, and it also leads to less empathy and guilt toward the targets of online conversations (Tian, 2009). Recent research reveals that due to the filtering of communication cues by online media, users tend to amplify their emotional reactions, creating an atmosphere in which exaggeration becomes the norm in communication (Caspi & Etgar, 2023). Syrjämäki et al. (2024) further found that compared to offline settings, individuals with difficulties in emotion regulation exhibit more pronounced disinhibitory characteristics online—visual anonymity weakens social norm constraints, leading to the externalization of negative emotions as aggressive remarks. Additionally, research in medical communication education has shown that online teaching, through chat functions, reduces students’ pressure to speak up and promotes open discussions (Ichikura et al., 2024). This enables students to dare to express confusion and dissatisfaction, which confirms the positive effect of anonymity on communication openness. However, it further verifies that online communication, due to visual anonymity, prompts individual “deindividuation”, weakening individuals’ perception of social ethics and norms. As a result, they are more willing to vent their instinctual energy and present themselves in their most primitive state, which can manifest as unrestrained abuse, insults, malicious attacks, and violations toward others (Li, 2020). In summary, we conclude that the lack of visual cues in online environments may weaken social constraints, exacerbating informational biases under negative attitudes (S. Zhou et al., 2023).

In the classic paradigm of social identity bias (SIB), the listener is virtual, and to some extent, communication in the current online environment bears a resemblance to this setup. When communicating online, such as through online chats or forum postings, people often express themselves to a virtual “listener”, and the real-time reactions of the recipient may be delayed or unclear, which is analogous to the lack of genuine interactive feedback in the classic paradigm. However, the online environment possesses its own unique characteristics, including a wide range and rapid speed of information dissemination, as well as diversified social platforms and communication modes. This makes the influence of the “virtual listener” in online communication more complex and diverse. Nonetheless, the classic paradigm provides a certain foundation for understanding communication in the online environment, and there exists a connection and comparability between the two. Both reflect, to varying degrees, the information transmission and cognitive processing processes of people in “indirect interaction” situations, which is of great significance for further exploring communication mechanisms.

### 2.3. Conceptual Model and Research Hypotheses

In the classic SIB paradigm, the speaker receives ambiguous information and an audience’s attitude, placing them in a situation of uncertainty or psychological conflict. This scenario or mindset enhances the speaker’s epistemic and relational motivations (Enestrom et al., 2024), driving them to create a shared reality with the audience (Hardin & Higgins, 1996). Once a shared reality is established between the speaker and the audience, the speaker regards the audience as a reliable source of information, and their individual behavior (description of information) and judgments (memory) are influenced by the audience’s attitudes, leading to the emergence of the SIB effect (Echterhoff & Higgins, 2019). Existing research has shown a positive correlation between the SIB effect and individuals’ self-reported levels of shared reality (Schmalbach et al., 2019). In the latest research by Enestrom et al. (2024), it was found that experiencing shared reality with close partners (i.e., perceiving an overlap in general internal states about the world) can reduce uncertainty about the environment, which in turn promotes meaning in work and life. Based the SIB effect, we propose the first two hypotheses of this study:

**H1.**  *People adjust their information descriptions according to the audience’s attitude. Specifically, when the audience’s attitude is positive, people will describe individuals positively; when the audience’s attitude is negative, people will describe individuals negatively.*

**H2.**  *The audience-tuning effect influences people’s memory orientation. Specifically, when the audience’s attitude is positive, people will recall individuals positively; when the audience’s attitude is negative, people will recall individuals negatively.*

Based on the social identity bias (SIB) effect and the theory of shared reality, we have incorporated the variable of communication modality and the factor of visual anonymity. Audience attitude and communication modality interactively influence the SIB effect, which encompasses two aspects: behavior (audience-tuning effect) and judgment (memory bias). In the online communication environment, communication modality exacerbates descriptive and memory biases under negative attitudes through visual anonymity, leading to enhanced “deindividuation”. Consequently, we propose Hypothesis 3:

**H3.**  *In online communication environments, people experience greater “deindividuation” due to visual anonymity, resulting in larger descriptive biases (audience-tuning effect) when holding negative attitudes, while the descriptive biases when holding positive attitudes are smaller compared to face-to-face communication.*

The memory bias caused by the audience-tuning effect within SIB has been confirmed by multiple studies. This reflects not only individuals’ active creation of shared reality but also the enduring impact of co-creating shared reality with others on individuals’ psychological representations (Higgins, 2019). Since memory serves as the foundation for other cognitive processes, memory bias is often used as a proxy indicator of successfully creating shared reality (Echterhoff et al., 2005). Furthermore, people are more sensitive to negative information and more likely to form memories of it (Erceg et al., 2025). Thus, we propose Hypothesis 4:

**H4.**  *Compared to offline communication, online communication results in larger memory biases under negative attitudes.*

**Figure 1:**
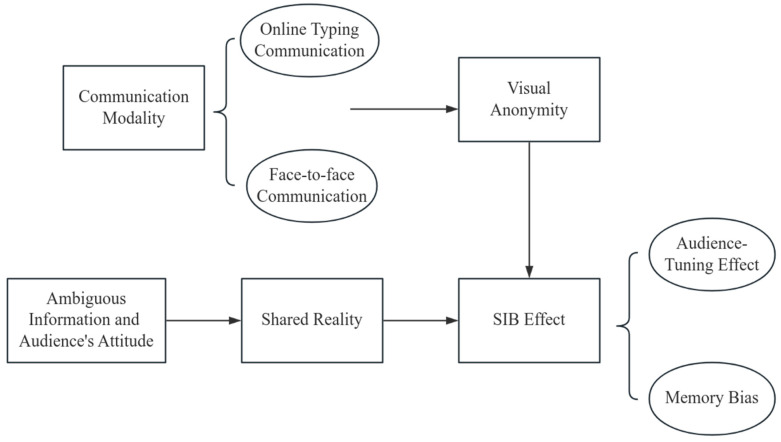
Conceptual model.

In conclusion, this study systematically reviews the literature and intermediary mechanisms of the SIB effect, combining the classic experimental paradigm of SIB. Building on previous theories, it aims to validate the SIB effect in the context of Chinese culture (Hypotheses 1 and 2). In addition, considering the background of information dissemination in the internet era and the unique characteristics of online communication compared to offline communication, this study aims to address the gap in cross-media comparisons within the SIB effect. It seeks to explore the impact of different communication modalities on the SIB effect (Hypotheses 3 and 4). This paper constructs a conceptual model based on the above (see Figure 1).

## 3. Methods

### 3.1. Participants

Referring to the most recent research on factors influencing the SIB effect (Wagner et al., 2024), a minimum of 68 participants was required to detect a medium-sized effect of *d* = 0.3 using G*Power (Faul et al., 2007) for power analysis with a statistical power of 0.80 and a Type I error probability of *alpha* = 0.05 (two-tailed). A total of 80 participants were recruited from Tianjin Normal University. Additionally, following previous research, a suspicion check (Echterhoff et al., 2008) was conducted after the experiment, where participants were asked to guess the purpose of the study and whether they believed the experimental setup (e.g., “Do you think the listener will read the message you sent?” “Do you trust the experimenter?”). Ten participants who expressed high suspicion about the study’s purpose or experimental setup were excluded. Ultimately, data from 70 participants (31 males) were included in the statistical analysis (see Table 1), with an average age of 20.17 ± 1.64 years. The sample was divided into two groups: a positive audience attitude group of 34 participants (15 males, average age 20.07 ± 1.03 years) and a negative audience attitude group of 36 participants (16 males, average age 19.69 ± 1.08 years). All participants were physically healthy, had no significant mental illnesses, had normal or corrected-to-normal vision, were native Chinese speakers, had no reading disabilities, and were right-handed. Prior to the experiment, all participants signed informed consent forms, and upon completion, each was paid CNY 20 by the experimenter as compensation. This study was approved by the ethics committee. Additionally, two experimenters (one male and one female) served as confederates, acting as listeners during the experiment, while participants served as speakers. The gender distribution between speakers and listeners was balanced.

### 3.2. Design

A two-factor mixed experimental design was employed, with audience’s attitude (positive vs. negative) as the between-subjects variable and communication modality (online vs. offline) as the within-subjects variable. The dependent variables were description valence, recall valence, description valence deviation, and recall valence deviation (see Table 2). Online communication was conducted by typing on the TC Lab platform, while offline communication took place face-to-face in the laboratory, with participants seated 50 cm apart (Schwartz et al., 2024). During the description phase, listeners were not allowed to provide verbal feedback to speakers but could express agreement or disagreement using facial expressions and gestures. To prevent interference from facial preference, the same confederate was used for same-gender participants, with gender balanced. Description valence refers to the rating of the information described by participants during the description phase, while recall valence refers to the rating of the original information recalled by participants about the target person during the free recall phase. After completing the entire experiment, two independent coders unrelated to the experiment rated the degree of positive or negative distortion in participants’ recalled information using a scale of −5 to +5, where +5 indicates the highest degree of positive distortion and −5 indicates the highest degree of negative distortion. A high degree of inter-coder agreement was required. Description deviation was calculated as the absolute difference between participants’ final description valence and the value of 0, and recall deviation was calculated as the absolute difference between participants’ final recall valence and the value of 0.

### 3.3. Materials

The ambiguous experimental materials selected for this study were developed based on English materials used in previous research (see Appendix A) (Echterhoff et al., 2005, 2017). The compilation process of the experimental materials was determined by referencing classic studies on the SIB experimental paradigm (Echterhoff et al., 2005, 2008; Higgins & Rholes, 1978; Zhang et al., 2025). The specific process is as follows. (1) Nomination Phase: Forty participants (18 males, average age 19.90 ± 1.12 years) were recruited to write ten pairs of synonymous adjective groups (one group with positive valence and one with negative valence) to describe college students. Examples include “straightforward and overly direct”, “independent and paranoid”, etc. Based on the adjectives with the highest nomination frequencies, an information item bank for the experimental materials was compiled. This bank included 14 vague behavioral statements, which could be interpreted positively or negatively. These 14 vague statements were organized into two sets of materials describing individual a and b, separately. For instance, “Once “a” makes up his mind to do something, it is as good as done no matter how long it might take or how difficult the going might be. Only rarely does he change his mind even when it might be better if he did”. This vague statement can be interpreted positively as “individual a is persistent” or negatively as “individual a is stubborn”. Both “persistent” and “stubborn” are adjectives with high nomination frequencies and opposite valences. It is important to note that the ambiguity of the information is crucial in the SIB experimental paradigm, as it allows participants to generate different attitudes toward the target individual. All information was controlled to be approximately 50 words in length. (2) Evaluation Phase: To verify the ambiguity of the experimental materials, before the formal experiment, the above information items were rated by 100 participants (49 males, average age 20.79 ± 2.01 years) who were unaware of the research purpose on a scale from −5 (very dislike) to +5 (very like). The final results showed that for each vague statement in the two sets of materials, less than 15% of the participants held a neutral attitude toward the target individual described in the paragraph. Among the remaining participants, the number of those who expressed liking or disliking the target individual described in the vague paragraph was roughly equal. This demonstrates that the experimental materials have good ambiguity (Zhang et al., 2025).

After the above compilation process, 14 vague statements (seven for individual a and seven for individual b) were finally obtained for use in the experiment. For example, for individual a, information 1 states: “It is very important for “a” to always be honest and direct to other people. When a friend showed him about his favorite work of art and asked him to comment, he mentioned that it needed to be worked on. (straightforward and overly direct)”. For individual b, information 1 states: “b” has many novel ideas and broad ideas, and can always find a new way to solve problems. He (or She) is not constrained by daily behavior norms, and he (or she) does not have goals and plans, so he (or she) often fails to complete tasks on time. (unrestrained and undisciplined)”. The remaining materials are provided in Appendix A. It should be noted that the content in parentheses for the above 14 statements will not be presented to the participants. The materials for individual a and individual b were used as materials for online and offline communication, respectively, with the order balanced during the experiment.

### 3.4. Procedure

**Figure 2:**
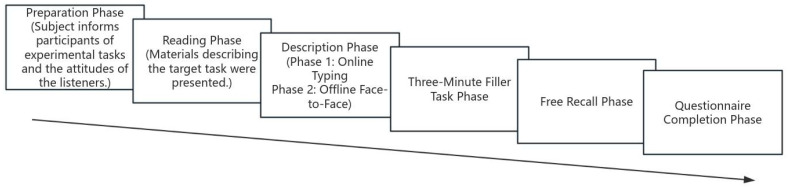
Experimental flowchart (applicable to both Phases 1 and 2).

The experiment was conducted in a quiet room. A standardized computer-based experimental procedure management workflow was designed using the TC Lab platform (Echterhoff et al., 2008) to guide participants through each step of the experiment and record their responses, with the experimenter providing necessary assistance at certain stages. Initially, both parties (the participant and the confederate) engaged in a brief introduction, where the experimenter introduced them to each other as participants and required both to sign an informed consent form, with the aim of convincing the real participant that the listener was also a participant and genuinely present. The experiment was conducted in two major phases (see Figure 2). Phase 1 is the Preparation Phase. The experimental instructions were presented using the TC Lab platform, informing participants that they would be participating in a study on “interpersonal communication and perception” (Higgins & Rholes, 1978; Echterhoff et al., 2005). Before reading the experimental materials, the experimenter explained that the listener had already completed a study involving 15 character materials, with one being the target character (individual a/b). The participant’s task was to enable the listener to identify the target character (individual a/b) based on their description and inadvertently inform the participant of the listener’s attitude (like or dislike) toward the target character (manipulation of the audience’s attitude). The participant’s task was to enable the listener to identify the target character from the 15 characters through their description. It begins with the Reading Phase. Participants viewed a short passage on the computer screen describing the behavior of the target character (individual a/b). This passage included seven ambiguous fragments, each capable of evoking a positive or negative trait label with equal likelihood (e.g., frugal vs. miserly). Next is the Description Phase (online condition). Participants were instructed to type a summary paragraph describing individual a and were told that this message would be simultaneously sent to the listener, who would perform the identification task. Participants were informed that they would not have any further contact with the listener during this stage, such as meeting again for mutual evaluation. Next is the Three-Minute Filler Task Phase. After the communication, participants completed a 3-min unrelated filler task. They were then informed that the listener had successfully identified individual a based on their description. The following is the Free Recall Phase. Participants were asked to recall the original text about individual a as accurately and in as much detail as possible and type it on the screen. The experimenter should explicitly emphasize that the recall should be of the original text presented on the screen, not the summary paragraph written by the participant. The next is the Questionnaire Survey. Participants were required to complete a series of questionnaires regarding their subjective psychological feelings about their communication partner, including the Generalized Shared Reality (SR-G, Rossignac-Milon et al., 2021) (see Appendix C), the Self-Report Questionnaire of Shared Reality (SR-T, Schmalbach et al., 2019; Rossignac-Milon et al., 2024) (see Appendix D), the Inclusion of the Other in the Self scale (IOS; Aron et al., 1991; Zhang et al., 2006) (see Appendix B), as well as questionnaires on embarrassment, suspicion, and so on. Phase 2 is the Preparation Phase. This phase was consistent with Phase 1, including the manipulation of the audience’s attitude. It begins with the Reading Phase. The requirements were the same as in Phase 1, but this time the materials described individual b/a. Next is the Description Phase (offline condition). Participants were instructed to communicate face-to-face with the listener to describe the target character (individual b/a) and were told that the listener would perform the identification task. They were informed that after this stage, they would need to fill out a questionnaire rating their impressions of each other and would be interviewed face-to-face about their communication. The confederate listener could not output new information during this stage but could respond appropriately to the participant’s output (based on the audience’s attitude). The following is the Three-Minute Filler Task Phase and Free Recall Phase. These phases were consistent with Phase 1. Next is the Questionnaire Survey. This was also consistent with Phase 1.

### 3.5. Data Analysis

The descriptive and recall information provided by the participants was coded by two independent coders who were unaware of the experimental design. The coding process is detailed as follows. (1) Information Disassembly: Based on the 14 descriptive statements in the experimental materials, the researchers disassembled the descriptive text into 14 information description units and the recall text into 14 information recall units. (2) Information Unit Evaluation: After studying the coding manual, including coding scales and examples, the two coders evaluated the valence of the information description units and information recall units. The information units were presented randomly, and a 11-point scale was used for scoring (−5 = very negative, 5 = very positive). The inter-coder reliability was sufficiently high. (3) Calculation of Information Valence: The mean score of the two coders was taken as the description valence and recall valence for each participant. IBM SPSS Statistics 26 and Mplus 8.0 software were used for statistical analysis in this study.

## 4. Results

### 4.1. Audience-Tuning Effect

For the inter-rater reliability test, the Pearson correlation coefficient between the scores of two independent raters, who were unrelated to the experiment, reached 90% (r = 0.986, *p* < 0.001), rendering their scores suitable for subsequent dependent variable analysis.

**Figure 3:**
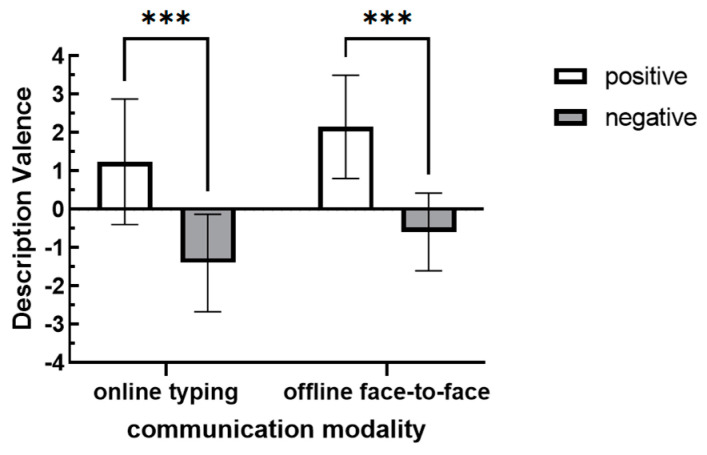
Description valence under different communication modalities and audience’s attitudes. (Note: *** indicates *p* < 0.001.).

A repeated measures ANOVA was conducted with communication modality and audience’s attitude as independent variables and description valence as the dependent variable. The results indicated (see Table 3) that there were significant differences in description valence across different communication modalities [*F*(1,68) = 16.620, *p* < 0.001, *η* = 0.20]. Specifically, the description valence during online communication (*M* = −0.12, *SD* = 1.96) was significantly lower than that during offline communication (*M* = 0.74, *SD* = 1.81). Furthermore, the audience’s attitude significantly influenced the participants’ description valence [*F*(1,68) = 127.947, *p* < 0.001, *η* = 0.65]. Participants in the negative audience’s attitude group exhibited significantly more negative description valence compared to those in the positive audience’s attitude group (*p* < 0.001) (see Figure 3). This confirmed the audience-tuning effect, supporting Hypothesis 1. People adjust their descriptive information valence according to audience’s attitude.

**Figure 4:**
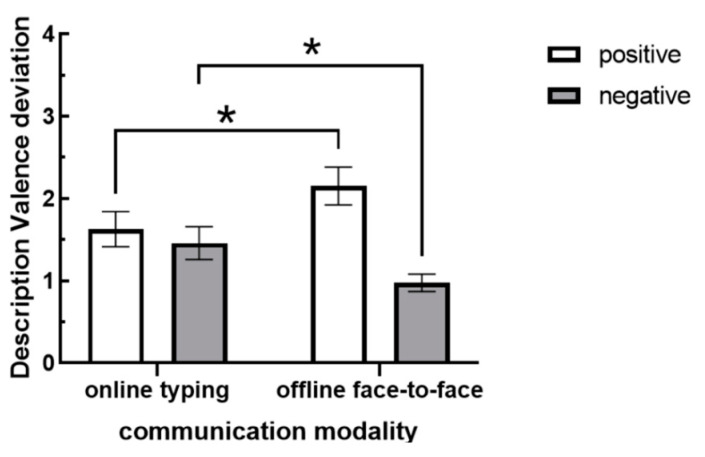
Description valence deviation under different communication modalities and audience’s attitudes (Note: * indicates *p* < 0.05).

Due to the inherent positivity and negativity of description valence, which precluded direct examination of interaction effects, the dependent variable was adjusted to “description valence deviation”, defined as the absolute difference between the description valence and the value of 0, to further test for interaction effects. The results (see Figure 4) revealed a significant interaction between communication modality and audience’s attitude [*F*(1,68) = 8.687, *p* = 0.004, *η* = 0.113]. Further simple effect tests showed that there was a significant difference in description valence deviation between online and offline communication modalities when the audience’s attitude was positive (*p* = 0.036). Specifically, the description valence deviation during online communication (*M* = 1.63, *SD* = 1.24) was significantly lower than that during offline communication (*M* = 2.15, *SD* = 1.34). Similarly, when the audience’s attitude was negative, there was also a significant difference in description valence deviation between online and offline communication modalities (*p* = 0.046). However, in this case, the description valence deviation during online communication (*M* = 1.46, *SD* = 1.20) was significantly higher than that during offline communication (*M* = 0.98, *SD* = 0.64). Hypothesis 3 was supported, suggesting that in online communication environments, people will be more likely to output negative information due to the greater “deindividuation” of visual anonymity, resulting in a greater amount of descriptive bias when it comes to negative attitudes (audience-tuning effect).

### 4.2. The Influence of the Audience-Tuning Effect on Memory

For the inter-rater reliability test, the Pearson correlation between the scores of two independent raters unrelated to the experiment reached 90% (*r* = 0.976, *p* < 0.001), indicating their scores can be used for subsequent dependent variable analysis.

**Figure 5:**
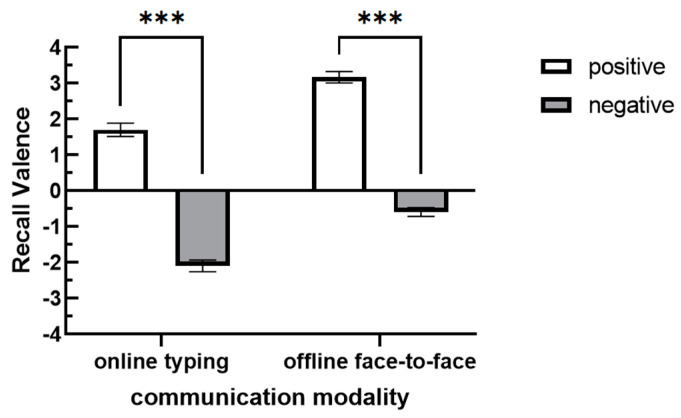
Recall valence under different communication modalities and audience’s attitudes. (Note: *** indicates *p* < 0.001.).

A repeated measures ANOVA was conducted with communication modality and audience’s attitude as independent variables and recall valence as the dependent variable. The results (see Table 4) showed that there were significant differences in recall valence across different communication modalities [*F*(1,68) = 94.973, *p* < 0.001, *η* = 0.58]. Specifically, the recall valence during online communication (*M* = −0.25, *SD* = 2.17) was significantly lower than that during offline communication (*M* = 1.24, *SD* = 2.06). Audience’s attitude significantly influenced participants’ recall valence [*F*(1,68) = 526.581, *p* < 0.001, *η* = 0.89]. Participants in the negative audience’s attitude group exhibited significantly more negative recall valence than those in the positive audience’s attitude group (*p* < 0.001) (see Figure 5). This validated the SIB effect, supporting Hypothesis 2. The audience-tuning effect also affects subsequent memory, leading to a recall bias in favor of the audience’s attitude toward the speaker.

**Figure 6:**
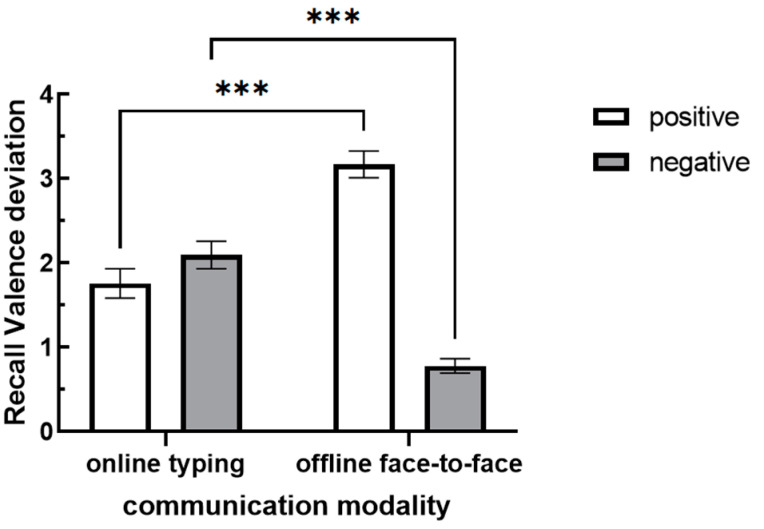
Recall valence deviation under different communication modalities and audience’s attitudes. (Note: *** indicates *p* < 0.001.)

Due to the positive and negative nature of recall valence, which prevents direct testing of interactions, the dependent variable was adjusted to “recall valence deviation”, defined as the absolute value of the difference between recall valence and the numerical value of 0, to further examine interactions. The results revealed (see Figure 6) a significant interaction between communication modality and audience’s attitude [*F*(1,68) = 87.148, *p* < 0.001, *η* = 0.562]. Further simple effect tests showed that there was a significant difference in recall valence deviation between online and offline communication modalities when audience’s attitude was positive (*p* < 0.001). Specifically, the recall valence deviation during online communication (*M* = 1.76, *SD* = 0.17) was significantly lower than that during offline communication (*M* = 3.17, *SD* = 0.13). When audience’s attitude was negative, there was also a significant difference in recall valence deviation between online and offline communication modalities (*p* < 0.001). However, in this case, the recall valence deviation during online communication (*M* = 2.10, *SD* = 0.17) was significantly higher than that during offline communication (*M* = 0.78, *SD* = 0.12). Hypothesis 4 was supported, demonstrating that after online communication produces a greater amount of description bias in negative attitudes compared to offline communication, it further leads to a greater amount of memory bias as well.

### 4.3. Shared Reality and IOS Score

To further elucidate the psychological mechanisms through which communication modality influences the SIB effect, we employed the Mplus 8.0 statistical software to conduct a bias-corrected Bootstrapping test (with 5000 samples) to examine the mediating effects of communication modality on memory bias. In the mediation analysis model of this study, different communication modalities (online and offline) were coded as dummy variables, while the mediator variables—IOS scores, generalized shared reality (SR-G), self-reported shared reality (SR-T), and the dependent variable recall valence deviation—were treated as continuous variables. The results of the mediation analysis are as follows. As shown in Table 5, the mediating effect of IOS was not significant, with an estimated value of 0.058 and a 95% confidence interval (CI) of [−0.007, 0.025]. Similarly, the mediating effect of SR-G was not significant (estimated value = 0.034, 95% CI = [−0.056, 0.158]), nor was that of SR-T (estimated value = 0.000, 95% CI = [−0.048, 0.064]). To further explore the relationships among these variables, we conducted a correlation analysis among communication modality, IOS scores, SR-G, SR-T, and recall valence deviation. The results, presented in Table 6, revealed significant correlations between communication modality and SR-G (*r* = 0.201, *p* < 0.05); IOS scores and SR-G (*r* = 0.467, *p* < 0.001), SR-T (*r* = 0.202, *p* < 0.01), and recall valence deviation (*r* = 0.187, *p* < 0.05); as well as between SR-G and SR-T (*r* = 0.524, *p* < 0.001). These findings suggest that IOS scores are closely related to shared reality, confirming their validity as an indicator of shared reality. Furthermore, the mediating mechanisms through which communication modality influences recall bias are not solely operated by the aforementioned mediator variables alone but involve more complex relationships that warrant further exploration.

## 5. Discussion

Using a mixed experimental design of 2 (audience’s attitude: positive/negative) × 2 (communication modality: online/offline), combined with the classic paradigm, this study uncovered the audience-tuning effect and its impact on memory, thereby validating the “saying-is-believing” (SIB) effect. Furthermore, the study identified differences in this effect across different communication modalities, with a focus on the characteristics of online communication. It explored the interaction between communication modality and audience attitude. The results revealed that under negative conditions, both descriptive bias and recall bias were greater in online compared to offline settings, whereas the opposite was true under positive conditions. Overall, online communication modality tends to facilitate the dissemination of negative information more than offline communication. This conclusion warrants further reflection.

Regarding the SIB effect, this paper proposes two hypotheses: individuals adjust their information descriptions based on the audience’s attitude, referred to as the “audience-tuning effect”, and this audience-tuning effect influences their memory direction, resulting in “memory bias”. These two influences pertain to individual behavior and judgment, respectively. The entire process is termed the “SIB effect”. The results support both hypotheses. Repeated measures ANOVA revealed that the audience’s attitude significantly affected description valence (*F*(1,68) = 127.947, *p* < 0.001, *η*^2^ = 0.65). Specifically, the description valence in the positive attitude group was significantly higher than that in the negative attitude group (*p* < 0.001), confirming that speakers indeed adjust their information expression based on the audience’s attitude, consistent with recent research by Lee et al. (2024) on the influence of the SIB effect on attitude change, where individuals adapt their language to construct a shared reality with the audience. Cognitive and relational motives jointly drive the SIB effect. Recent research in this area by Liang et al. (2021) found that speakers’ high epistemic trust in listeners, or trust in group judgments, facilitates the SIB effect. Building on cognitive trust, Wagner et al. (2024) identified the role of cognitive accessibility, where the creation of a shared reality with the audience triggers a fundamental cognitive mechanism that facilitates the retrieval of information about audience-consistent (vs. audience-inconsistent) traits of the target individual. Consistent with the conceptual model in the introduction, this suggests that the intake of ambiguous information and the audience’s attitude motivates speakers to create a shared reality with the audience, both cognitively and relationally, leading to descriptive biases. This aligns with Enestrom et al.’s (2024) research, which found that constructing a shared reality with others reduces individual uncertainty, referring here to ambiguous information and “unintentionally” informed audience attitudes. Additionally, the results also found a significant main effect of the audience’s attitude on recall valence (*F*(1,68) = 526.581, *p* < 0.001, *η*^2^ = 0.89), with the recall valence in the positive attitude group being significantly higher than that in the negative attitude group (*p* < 0.001), further formalizing that the construction of a shared reality persistently affects memory representations (Sarasso et al., 2024). In summary, the SIB effect found in this study within the Chinese cultural context aligns with previous research findings on the SIB effect in sexual harassment scenarios (Pierucci et al., 2014), collectivist-individualist cultural contexts (Skorinko et al., 2015), and German-Turkish national contexts (Echterhoff et al., 2017). This further demonstrates the cross-cultural consistency of the SIB effect.

Regarding the impact of communication modality on the SIB effect, this paper proposes two additional hypotheses. In online communication environments, individuals are more likely to experience “deindividuation” due to visual anonymity, leading to a greater tendency to convey negative information. Consequently, when holding negative attitudes, there is a larger descriptive bias, which corresponds to a larger memory bias. Our research findings reveal that the overall description valence is lower in online communication (*M* = −0.12 vs. *M* = 0.74 for offline), and similarly, the recall valence of information is also lower in online communication (*M* = −0.25 vs. *M* = 1.24 for offline). This indicates that there are indeed differences between online and offline communication, which to some extent corroborates Caspi and Etgar’s (2023) conclusion that visual anonymity amplifies negative expressions, reflected in the more negative valence of online communication compared to offline. Furthermore, we changed the dependent variables to “description valence bias” and “recall valence bias” to further explore the interaction between communication modality and audience’s attitude. The results found a significant interaction effect (*F*(1,68) = 8.687, *p* = 0.004). Specifically, under negative attitudes, the descriptive bias is larger in online communication (*M* = 1.46 vs. *M* = 0.98 for offline), while under positive attitudes, the online bias is lower (*M* = 1.63 vs. *M* = 2.15 for offline). This validates Hypothesis 3. This suggests that online communication may lead to moral disengagement, as evidenced by Chen and Chen’s (2025) recent research on online moral disengagement, which indicates that individuals’ moral norms may weaken when communicating online, leading them to behave in ways they would not in real life. The results also support Mylek et al.’s (2024) “social-compensation hypotheses”, which proposes that online communication compensates for real-life social deficiencies, such as the impact of emotions like depression and anxiety on offline interactions, serving as a compensatory mechanism for individuals under stress. In the online condition of this study, due to visual anonymity, people were more “bold” in describing the target individual’s shortcomings to strangers. In contrast, in offline communication, people may be more inclined to convey positive information to quickly establish friendly relationships with others for self-protection purposes (Hutchison et al., 2024). Additionally, the results of this study show that the recall bias in the online negative attitude group is significantly higher than that in the offline group (*M* = 2.10 vs. *M* = 0.78, *p* < 0.001). This can be understood from the perspective of the audience-tuning effect leading to memory bias, or from the perspective that people are more sensitive to negative information and more likely to form memories of it (Erceg et al., 2025).

Furthermore, this study found that the mediating role of shared reality (SR-G, SR-T) in the impact of communication modality on the SIB effect was not significant. This may be due to the lack of non-verbal cues in online interactions, which weakened the perception of shared reality (Xu et al., 2024). This also validates the conceptual model of this study, where communication modality and audience’s attitude influence the SIB effect through different mechanisms. Regarding communication modality, we further explain its mechanisms from the perspective of the characteristics of online communication, namely visual anonymity. Additionally, recent research has found that, besides conveying negative information, the characteristics of online communication lead individuals to engage in more behaviors online that they would not do offline, which is also reflected in the normativeness and innovation of the information conveyed. Wang et al. (2025) discovered the role of information normativeness in online healthcare consultations, which are a typical example of complex human-to-human communication requiring both effectiveness and efficiency. Therefore, adopting online consultations facilitates individuals’ understanding of consultation information. As for innovation, it plays a significant role primarily in the field of enterprise management (Farte et al., 2025).

Nevertheless, this experiment still has some limitations. Firstly, this study lacks an in-depth exploration of the deeper underlying mechanisms influencing the SIB effect. For instance, how does the visual anonymity of online communication, compared to offline communication, differ in terms of individuals’ epistemic motivation (Kopietz et al., 2010) to understand the truth of the world, their relational motivation (Pierucci et al., 2013) to establish social connections, and their perception of epistemic authority (Echterhoff et al., 2017). Secondly, the classification of communication modality in this study is not detailed enough, for example, distinguishing between online typing and online video/audio communication, as well as between offline face-to-face and back-to-back communication. Thirdly, this study only examines the one-way process of the listener’s influence on the speaker. The study was conducted in a laboratory setting that inherently cannot explore the speaker’s influence on the listener or the mutual influence between the communicating parties, resulting in low external validity of the research results. Future studies could build upon the classic SIB experimental paradigm, using eye-tracking devices under conditions of a quiet environment and fixed social distance to record eye contact between speakers and listeners, examining the SIB effect in real interactive environments. This approach would enhance the external validity of SIB research.

## 6. Conclusions

This study has discovered the cross-media universality of the SIB effect, indicating that people adjust their individual descriptions and recollections based on the audience’s attitude in both online and offline environments. When the audience’s attitude is positive, individuals tend to describe and recall individuals positively. Conversely, when the audience’s attitude is negative, descriptions and recollections become negative. This is the SIB effect. Furthermore, communication modality interacts with the audience’s attitude to influence the SIB effect. When the audience’s attitude is positive, offline communication exhibits a greater deviation in description and recall valence compared to online communication, making the SIB effect more pronounced. Conversely, when the audience’s attitude is negative, online communication shows a greater deviation in description and recall valence compared to offline communication, also enhancing the SIB effect. Overall, online communication is more likely to convey negative information than offline communication. This study holds certain theoretical and practical significance.

### 6.1. Theoretical Significance

In terms of theory, this study enriches and expands the field of SIB by conducting the first cross-media exploration of the SIB effect. It identifies the interactive influence of communication modality and audience’s attitude on the SIB effect, revealing that the SIB effect is more pronounced offline compared to online under positive conditions, and more pronounced online compared to offline under negative conditions. Furthermore, this study extends the classic SIB paradigm to real-life interaction contexts, examining related issues of the SIB effect within the actual temporal and spatial dimensions. Additionally, it constructs a conceptual model based on shared reality and expands shared reality theory by first verifying the interference of visual anonymity in online environments on the construction of shared reality, providing a new perspective for interpersonal communication theory in the digital age. Furthermore, this study conducts a deeper analysis of online communication, combining previous research to propose that visual anonymity in online communication exacerbates the amplification effect of audience’s attitude differences on description bias and memory bias through “deindividuation”. In summary, this study makes theoretical contributions to the field of SIB and media communication.

### 6.2. Practical Significance

In terms of practice, this study further enhances our in-depth understanding of both online and offline communication modalities. Choosing the appropriate communication modality in different situations has practical significance for the effectiveness of work and learning (such as memory) and encourages people to consciously discern various opinions in the internet environment, which is crucial for improving the online environment. In addition, this study also offers guidance for businesses. For employees, they should be vigilant about the amplifying effect of visual anonymity on negative expressions in online communication, and they can adopt a “cooling-off period” strategy (such as delaying email sending) to comprehensively analyze issues and reduce impulsive remarks. Meanwhile, before discussing sensitive topics offline, they can use outlines and simulated dialogues to reduce the risk of being easily influenced by others’ attitudes. For managers, they need to flexibly select communication modalities based on the context—for sensitive topics such as performance feedback, offline face-to-face communication is preferred to capture non-verbal cues, while online communication should combine emojis and structured language (such as the “sandwich feedback method”: affirmation-suggestion-support) to balance the drawbacks of anonymity. For organizations, they should build intelligent hybrid communication platforms that allow anonymous expression in scenarios such as opinion solicitation, but require mandatory real-name use in collaborative decision-making. These platforms should also incorporate AI real-time emotion monitoring (e.g., recognizing aggressive vocabulary to trigger popup reminders) and post-event analysis functions (generating emotion reports to assist in management interventions). Meanwhile, using online-offline linkage mechanisms (such as anonymous feedback → offline focus groups → public improvement loops), organizations can ensure both communication efficiency and psychological safety. In summary, communication is essential in enterprises, and choosing the appropriate communication modality based on different scenarios is particularly important, as it directly affects individual behavior and judgment within the enterprise and even the future of the entire enterprise.

## Figures and Tables

**Table 1:** Participants’ demographic data.

	Totals	Male	Female	Average Age (M ± SD)
Total Participants	70	31	39	20.17 ± 1.64
Positive Audience Attitude Group	34	15	19	20.07 ± 1.03
Negative Audience Attitude Group	35	16	20	19.69 ± 1.08

**Table 2:** Measured variable items.

Measured Variable	Measurement Methods
Description Valence	Rating of the information described by participants during the description phase
Recall Valence	Rating of the original information recalled by participants about the target person during the free recall phase
Description Valence Deviation	Absolute difference between participants’ final description valence and the value of 0
Recall Valence Deviation	Absolute difference between participants’ final recall valence and the value of 0

**Table 3:** Information description valence (M ± SD) under different communication modalities and audience’s attitudes.

Audience’s Attitude	Online Typing Communication	Face-to-Face Communication
Positive (n = 34)	1.242 ± 1.638	2.152 ± 1.346
Negative (n = 36)	−1.400 ± 1.268	−0.600 ± 1.013

**Table 4:** Information recall valence (M ± SD) under different communication modalities and audience’s attitudes.

Audience’s Attitude	Online Typing Communication	Face-to-Face Communication
Positive (n = 34)	1.704 ± 1.103	3.170 ± 0.919
Negative (n = 36)	−2.095 ± 0.974	−0.588 ± 0.729

**Table 5:** Results of the mediation analysis on the effects of shared reality and IOS scores on recall valence deviation.

Mediation Effect Pathway	Estimated Value	Estimated Value 95% CI	
		Low	High
Communication Modality → IOS Score → Recall Valence Deviation	0.058	−0.007	0.025
Communication Modality → SR-G → Recall Valence Deviation	0.034	−0.056	0.158
Communication Modality → SR-T → Recall Valence Deviation	0.000	−0.048	0.064

**Table 6:** Correlations among communication modality, IOS scores, generalized shared reality, self-reported shared reality, and recall valence deviation.

Variables	1	2	3	4	5
Communication Modality	1				
IOS Score	0.155	1			
SR-G	0.201 *	0.467 ***	1		
SR-T	0.054	0.202 **	0.524 ***	1	
Recall Valence Deviation	0.000	0.187 *	0.082	0.003	1

Note: * indicates *p* < 0.05, ** indicates *p* < 0.01, and *** indicates *p* < 0.001.

**Figure A1:**
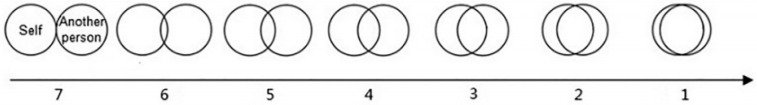
Self-Other Overlap (IOS).

## Data Availability

Due to the nature of this research, participants of this study did not agree for their data to be shared publicly, so supporting data are not available.

## Appendix A. Materials

a

It is very important for “a” to always be honest and direct to other people. When a friend showed him about his favorite work of art and asked him to comment, he mentioned that it needed to be worked on.

“a” attached great importance to social moral standards and personal codes of conduct. While working in the company, he would tell on colleagues who violate the code and regulations for personal gain, even about something trivial.

Once “a” makes up his mind to do something, it is as good as done no matter how long it might take or how difficult the going might be. Only rarely does he change his mind even when it might be better if he did.

“a” knows his strengths and tends to solve problems independently. He is not easy to accept other people’s opinions and suggestions and never changes his mind, even if sometimes the result is better in another way.

“a” always tries to save money. He usually chooses the cheapest item on the menu when going out to eat with friends. He often buys clothes and other daily necessities at discounts in shops to avoid unnecessary expenses.

“a” is a person who speaks English well and has read many books about English grammar and communication. He can talk about various interesting topics fluently in English. When talking with others in English, he always likes to correct other people’s grammatical mistakes.

“a” recently started making attempts to keep up to date with cultural knowledge. He read a book about Europe, sat in a music appreciation workshop, and eats in fashionable ethnic restaurants. When being with friends, he often talks at length about foreign cultures and art.

b

“b” is frank and outspoken. When it comes to things, he (or she) says what he (or she) thinks directly and does not hold back. When he (or she) disagrees with others, he (or she) always fights back without considering the situation or the feelings of others.

When ordering food for a dinner with friends, he (or she) would order it even if it was enough for everyone to eat, and then offer to pay for it afterwards. He (or She) always buys things he (or she) does not need just because he (or she) is interested in them, regardless of their practical value.

“b” has many novel ideas and broad ideas and can always find a new way to solve problems. He (or She) is not constrained by daily behavior norms, and he (or she) does not have goals and plans, so he (or she) often fails to complete tasks on time.

In daily life, he (or she) takes the initiative to help classmates take the express, when someone is in trouble, he (or she) always lends a hand. Even if the other person is not willing to do so, he (or she) will provide them with a solution he (or she) thinks is appropriate and urge them to complete it.

He (or She) is good at communication and understands a variety of topics. He (or She) can be the leader of the topic in any occasion and can talk with everyone. Sometimes, he (or she) would use other people’s personal issues as a topic to gain attention, and sometimes he (or she) would make fun of others in public, with no regard for others’ feelings.

Note: In the Appendix, the English materials introducing ‘a’ are from Echterhoff et al. (2005, 2008), and their corresponding Chinese translations are from Zhang et al. (2025). For the comparable ambiguous materials introducing ‘b’, both the English ver-sions and their corresponding Chinese translations are from Zhang et al. (2025).

## Appendix B. Self-Other Overlap (IOS)

In the picture, there are seven pairs of circles with different overlaps, and the overlapping parts of the circles represent the closeness between you and your partner. The more overlaps there are, the closer you are. Please select the pair of circles that best describes your relationship and then select the corresponding serial number.

## Appendix C. Generalized Shared Reality (SR-G)

(7-point rating, from 1 = Strongly disagree to 7 = Strongly agree)

(1) We frequently think of things at the exact same time.

(2) Through our discussions, we often develop a joint perspective.

(3) We typically share the same thoughts and feelings about things.

(4) Events feel more real when we experience them together.

(5) The way we think has become more similar over time.

(6) We often anticipate what the other is about to say.

(7) We are more certain of the way we perceive things when we are together.

(8) We often feel like we have created our own reality.

## Appendix D. Shared Reality About a Target (SR-T)

(7-point rating, from 1 = Strongly disagree to 7 = Strongly agree)

(1) I think that Liu and I are on the same wavelength with regard to a/b.

(2) I feel the same way about a/b as Liu does.

(3) I agree with Liu‘s point of view of a/b.

(4) I agree with Liu‘s point of view of a/b.

(5) I agree with Liu‘s perception of a/b.
