# Correction: Yin and Liu (2025). The Influence of Communication Modality on the “Saying-Is-Believing” Effect. *Behavioral Sciences*, *15*(5), 639

**DOI:** 10.3390/bs15111446

**Published:** 2025-10-24

**Authors:** Rui Yin, Xianyun Liu

**Affiliations:** 1 Faculty of Psychology, Tianjin Normal University, Tianjin 300387, China; 2230340024@stu.tjnu.edu.cn; 2 Key Research Base of Humanities and Social Sciences of the Ministry of Education, Academy of Psychology and Behavior, Tianjin Normal University, Tianjin 300387, China; 3 Tianjin Key Laboratory of Student Mental Health and Intelligence Assessment, Tianjin 300387, China

**Missing Citation**

In the original publication (Yin & Liu, 2025), Zhang, H., Cui, Z., Wagner, U., & Echterhoff, G. (2025). *The influence of romantic relationships on the “saying is believing” effect*. Available online: https://osf.io/yq2z5/ (accessed on 23 May 2023) was not cited. The citation has now been inserted in 3. Methods, 3.3. Materials, Paragraph 1 and should read as follows:

The ambiguous experimental materials selected for this study were developed based on English materials used in previous research (see Appendix A) (Echterhoff et al., 2005, 2017). The compilation process of the experimental materials was determined by referencing classic studies on the SIB experimental paradigm (Echterhoff et al., 2005, 2008; Higgins & Rholes, 1978; Zhang et al., 2025). The specific process is as follows. (1) Nomination Phase: Forty participants (18 males, average age 19.90 ± 1.12 years) were recruited to write ten pairs of synonymous adjective groups (one group with positive valence and one with negative valence) to describe college students. Examples include “straightforward and overly direct”, “independent and paranoid”, etc. Based on the adjectives with the highest nomination frequencies, an information item bank for the experimental materials was compiled. This bank included 14 vague behavioral statements, which could be interpreted positively or negatively. These 14 vague statements were organized into two sets of materials describing individual a and b, separately. For instance, “Once “a” makes up his mind to do something, it is as good as done no matter how long it might take or how difficult the going might be. Only rarely does he change his mind even when it might be better if he did”. This vague statement can be interpreted positively as “individual a is persistent” or negatively as “individual a is stubborn”. Both “persistent” and “stubborn” are adjectives with high nomination frequencies and opposite valences. It is important to note that the ambiguity of the information is crucial in the SIB experimental paradigm, as it allows participants to generate different attitudes toward the target individual. All information was controlled to be approximately 50 words in length. (2) Evaluation Phase: To verify the ambiguity of the experimental materials, before the formal experiment, the above information items were rated by 100 participants (49 males, average age 20.79 ± 2.01 years) who were unaware of the research purpose on a scale from −5 (very dislike) to +5 (very like). The final results showed that for each vague statement in the two sets of materials, less than 15% of the participants held a neutral attitude toward the target individual described in the paragraph. Among the remaining participants, the number of those who expressed liking or disliking the target individual described in the vague paragraph was roughly equal. This demonstrates that the experimental materials have good ambiguity (Zhang et al., 2025).

**Text Correction**

There was an error in the original publication—**Lack of Acknowledgements**.

A correction has been made to the **Acknowledgements**:

We sincerely thank Huan Zhang (Tianjin Normal University), Ullrich Wagner (University of Muenster), and Gerald Echterhoff (University of Muenster) for their invaluable contributions to the experimental materials, selection of research topics, and other aspects. We are also grateful to all participants for their involvement in this study. Finally, we thank the editor and anonymous reviewers for their insightful feedback and careful evaluation of the manuscript.

There was an error in the original publication. Add a note after Appendix A. Materials, part b.

A correction has been made to Appendix A. Materials, part b:

b

“b” is frank and outspoken. When it comes to things, he (or she) says what he (or she) thinks directly and does not hold back. When he (or she) disagrees with others, he (or she) always fights back without considering the situation or the feelings of others.

When ordering food for a dinner with friends, he (or she) would order it even if it was enough for everyone to eat, and then offer to pay for it afterwards. He (or She) always buys things he (or she) does not need just because he (or she) is interested in them, regardless of their practical value.

“b” has many novel ideas and broad ideas and can always find a new way to solve problems. He (or She) is not constrained by daily behavior norms, and he (or she) does not have goals and plans, so he (or she) often fails to complete tasks on time.

In daily life, he (or she) takes the initiative to help classmates take the express, when someone is in trouble, he (or she) always lends a hand. Even if the other person is not willing to do so, he (or she) will provide them with a solution he (or she) thinks is appropriate and urge them to complete it.

He (or She) is good at communication and understands a variety of topics. He (or She) can be the leader of the topic in any occasion and can talk with everyone. Sometimes, he (or she) would use other people’s personal issues as a topic to gain attention, and sometimes he (or she) would make fun of others in public, with no regard for others’ feelings.

Note: In the Appendix, the English materials introducing ‘a’ are from Echterhoff et al. (2005, 2008), and their corresponding Chinese translations are from Zhang et al. (2025). For the comparable ambiguous materials introducing ‘b’, both the English versions and their corresponding Chinese translations are from Zhang et al. (2025).

**References**

With this correction, the order of some references has been adjusted accordingly.

The authors state that the scientific conclusions are unaffected. This correction was approved by the Academic Editor. The original publication has also been updated.
